# The relationship between applied energy and ablation zone volume in patients with hepatocellular carcinoma and colorectal liver metastasis

**DOI:** 10.1007/s00330-017-5266-1

**Published:** 2018-03-13

**Authors:** Wouter J. Heerink, A. Millad Solouki, Rozemarijn Vliegenthart, Simeon J. S. Ruiter, Egbert Sieders, Matthijs Oudkerk, Koert P. de Jong

**Affiliations:** 1 Department of Radiology, University of Groningen, University Medical Center Groningen, Hanzeplein 1, 9713 GZ Groningen, The Netherlands; 2 Center for Medical Imaging North East Netherlands, University of Groningen, University Medical Center Groningen, Groningen, The Netherlands; 3 Department of HPB Surgery, University of Groningen, University Medical Center Groningen, Groningen, The Netherlands

**Keywords:** Ablation techniques, Carcinoma, Hepatocellular, Radiology, Interventional, Multidetector computed tomography, Liver diseases

## Abstract

**Objectives:**

To study the ratio of ablation zone volume to applied energy in computed tomography (CT)-guided radiofrequency ablation (RFA) and microwave ablation (MWA) in patients with hepatocellular carcinoma (HCC) in a cirrhotic liver and in patients with colorectal liver metastasis (CRLM).

**Methods:**

In total, 90 liver tumors, 45 HCCs in a cirrhotic liver and 45 CRLMs were treated with RFA or with one of two MWA devices (MWA_A and MWA_B), resulting in 15 procedures for each tumor type, per device. Device settings were recorded and the applied energy was calculated. Ablation volumes were segmented on the contrast-enhanced CT scans obtained 1 week after the procedure. The ratio of ablation zone volume in milliliters to applied energy in kilojoules was determined for each procedure and compared between HCC (*R*_HCC_) and CRLM (*R*_CRLM_), stratified according to ablation device.

**Results:**

With RFA, *R*_HCC_ and *R*_CRLM_ were 0.22 mL/kJ (0.14–0.45 mL/kJ) and 0.15 mL/kJ (0.14–0.22 mL/kJ; *p* = 0.110), respectively. With MWA_A, *R*_HCC_ was 0.81 (0.61–1.07 mL/kJ) and *R*_CRLM_ was 0.43 (0.35–0.61 mL/kJ; *p* = 0.001). With MWA_B, *R*_HCC_ was 0.67 (0.41–0.85 mL/kJ) and *R*_CRLM_ was 0.43 (0.35–0.61 mL/kJ; *p* = 0.040).

**Conclusions:**

With RFA, there was no significant difference in energy deposition ratio between tumor types. With both MWA devices, the ratios were higher for HCCs. Tailoring microwave ablation device protocols to tumor type might prevent incomplete ablations.

**Key Points:**

*• HCCs and CRLMs respond differently to microwave ablation*

*• For MWA, CRLMs required more energy to achieve a similar ablation volume*

*• Tailoring ablation protocols to tumor type might prevent incomplete ablations*

## Introduction

For over 15 years, hepatic malignancies have been treated successfully with radiofrequency ablation (RFA) and microwave ablation (MWA) [1–3]. The most important drawback of thermoablative therapies is recurrence of disease at the ablation site, with reported recurrence rates of 5.0–32.1% [2, 4–6]. Independent risk factors for incomplete ablation and ablation site recurrence are larger tumor size, proximity of peritumoral vessels, improper placement of the ablation needle, and insufficient safety margin around the liver tumor [7–9].

To ensure complete coverage of the liver tumor including a safety margin, the creation of a predictable ablation zone is crucial. Ablation protocols provided by manufacturers are mostly based on ex vivo ablation of nonperfused, nondiseased livers from animals. It can be expected that the resulting ablation zones in these livers would differ significantly from ablation zones after in vivo treatment of diseased livers in humans. Studies investigating the reproducibility and reliability of RFA and MWA in different tumor types and in abnormal underlying liver parenchyma (cirrhosis) in humans are lacking.

We designed this study to evaluate the ablation zone volume after RFA and MWA. The aims of this study were: (1) to find the relationship between the amount of applied energy and the resulting ablation zone volume for RFA and MWA devices during in vivo ablation in patients, and (2) to investigate whether the ratio of ablation zone volume in milliliters to applied energy in kilojoules [*R*(AZ:E)] differs between hepatocellular carcinoma (HCC) in a cirrhotic liver and colorectal liver metastasis (CRLM).

## Materials and methods

### Patients

The study was approved and the need for informed consent was waived by the Institutional Review Board of the University Medical Center Groningen (no. 2015/521). Data were processed anonymously. Thermoablation of liver tumors was introduced in 2000 in our hospital, ultrasound-guided during open surgery and CT-guided during percutaneous procedures. Over 600 ablation procedures (approximately 70% percutaneously) have been performed for various types of liver tumors in our hospital. In the present study we analyzed only patients who underwent percutaneous CT-guided thermoablation for either CRLM or HCC in the period from March 2009 to January 2016. All patients were discussed in a tumor board meeting in which the decision for percutaneous thermoablation was made.

Patients were included in this study if they were treated for HCC in a cirrhotic liver or for CRLM. Patients were excluded if: (1) there was overlap between the ablation zones of multiple lesions, (2) the tumor had previously been treated with ablation therapy, or (3) surgical clips caused beam hardening on the control CT images preventing adequate segmentation of the ablation zone. For each of the three ablation devices used, 30 ablation treatments were included: 15 tumors in patients consecutively treated for HCC, and 15 tumors in patients consecutively treated for CRLM. In this paper we adhere to the standard terminology and reporting criteria recommended by Ahmed et al. [10].

### Procedures

Procedures were performed by one of two surgeons (K. de J. and E.S.) with 16 and 12 years of experience in liver ablation, respectively, with the support of a radiologist. All procedures were performed under general anesthesia. Procedural CT scans and needle manipulations were performed during maximal expiration and after full elastic recoil of the thorax. This situation was obtained when CO_2_ monitoring of the exhaled breathing air in the respiratory tube revealed a completely flat baseline. Thermoablation was performed with one of three devices (see below), and manufacturers’ protocols were followed. Device settings were recorded during the procedures. Needle placement was performed based on the expected ablation zone size described by the manufacturer, considering a sufficient (>5 mm) safety margin around the tumor. Larger lesions were treated by creating several partially overlapping ablation zones. After treatment of the tumor, the ablation needle was removed while ablating the tract.

### CT scan protocol

Control CT scans were acquired 1 week after the ablation procedure on a 64-multidetector CT system (Somatom Sensation 64; Siemens Medical, Erlangen, Germany). The tube voltage was 120 kVp and the quality reference tube current was 120 mAs. Scans were acquired prior to administration of intravenous contrast agent (110 mL of Iomeron 300; Bracco Imaging), and in the arterial and portal venous phases, and reconstructed with slice thicknesses of 2–5 mm, 0.75–3 mm and 2 mm using a medium smooth B30f kernel.

### Ablation systems

The RFA system consisted of a 480-kHz generator with a maximum power output of 250 W (RF 3000 generator with Leveen needles; Boston Scientific Corp., Natick, MA). The diameters of the RFA needle electrodes with umbrella arrays ranged from 2 to 5 cm. The diameter of the needle electrodes was determined by the size of the tumor.

MWA system A consisted of a 2.45-GHz generator generating a maximum of 140 W with water-cooled needle electrodes (Acculis Sulis VpMTA; Microsulis Medical, Denmead, UK). MWA system B also used a 2.45-GHz frequency band, generating a maximum of 100 W, with water-cooled needles and thermal, field, and wavelength control to optimize the predictability of the ablation zone (Emprint MWA Generator; Covidien, Dublin, Ireland).

### Ablation protocols and applied energy

The manufacturer of the RFA system provided separate algorithms for each of the various needle diameters, prescribing the time and power settings to be applied during the procedure. The power of the system was manually increased according to this algorithm until roll-off was achieved. Roll-off is defined as a steep rise in tissue impedance, and is an indication of a successful ablation cycle. For each tumor, two complete ablation cycles were generated according to the manufacturer’s algorithm. During the RFA procedure, the time required to achieve roll-off and the roll-off indication power were recorded for each ablation cycle. These measurements were used to determine the area under the curve for time and power, resulting in the amount of energy applied in each ablation cycle. Next, the total amount of applied energy to each tumor was determined by summing the applied energy of all ablation cycles.

The manufacturers of MWA systems A and B provided tables with specific power and time settings according to the expected resulting ablation size. Appropriate settings were chosen, and these were recorded during the procedures. The power and time settings were used to determine the amount of applied energy in each ablation cycle, and subsequently summed per lesion to determine the total amount of energy applied.

### Ablation zone volumetry

Volumetry of the ablation zone was performed on the portal venous phase acquisition with 2-mm slice reconstruction using the semiautomatic Liver Lesion Segmentation tool (MM Oncology package; *syngo*.via; Siemens Medical, Erlangen, Germany). After initial manual measurement of the cross section of the tumor, this tool automatically delineated the tumor, and this was subsequently verified for each slice and, when necessary, corrected by two independent observers (W.H. and A.S.). Interobserver reliability of volumetry was expressed in terms of the intraclass correlation coefficient (ICC). An ICC value larger than 0.90 was considered as high agreement. In ten ablation volumes with a discrepancy in delineation between the observers of more than 10%, agreement in delineation between the ablation zone and perilesional enhancement was checked in a consensus meeting of the two observers, and was subsequently obtained by consensus. For the other ablation zones, the mean of the ablation zone volumes obtained by the observers was used for statistical analysis. *R*(AZ:E) was determined by dividing the ablation zone volume in milliliters by the amount of energy in kilojoules applied in each procedure.

Tumor size was determined as the largest diameter on contrast-enhanced transverse CT images acquired minutes before the ablation procedure. Tumor volume as a percentage of the total ablation zone volume was calculated by dividing the estimated tumor volume (based on diameter, assuming the lesions were spherical) by the total ablation zone volume. Ablations were considered incomplete when the control CT scan acquired after 1 week revealed that the ablation zone did not completely cover the tumor including a margin of at least 5 mm around the tumor in all directions. The potential for tumors to show the heat-sink effect was categorized as ‘high’ or ‘low’ depending on the peritumoral vascularity. The potential was considered to be high if tumor-abutting vessels with a diameter of ≥3 mm were present, as defined previously [8].

### Statistical analysis

Patient age, lesion size, peritumoral vascularity, ablation time, energy applied, number of ablation needle positions, ablation zone volume, energy deposition ratio, tumor percentage in ablation zone volume, and number of incomplete ablations were compared between the three ablation devices and between tumor types stratified according to the ablation device. *R*(AZ:E) was compared between tumor types using the Mann-Whitney *U* test. For each device, the *R*(AZ:E) of tumors with a potentially high and low heat-sink effect were compared. Potential correlations between peritumoral vascularity and tumor type were evaluated using the chi-squared test. For RFA, the effect of the size of the needle’s umbrella array on *R*(AZ:E) was evaluated using Spearman’s rank test, and median array diameters were compared between HCC and CRLM. The *R*(AZ:E) of CRLMs were compared between those who did and did not receive prior systemic therapy.

Parameters were tested for normality using the Shapiro-Wilk test. The means and standard deviations (SD) of continuous, normally distributed parameters were determined and compared using one-way analysis of variance or the independent-samples *t* test. The medians and interquartile ranges (IQR) of non-normally distributed variables were determined and tested for homogeneous non-normality and compared using the Kruskal-Wallis test or the Mann-Whitney *U* test. Dichotomous data were compared using Fisher’s exact test. Differences with *p* values <0.05 were considered significant, and *p* values were not adjusted for multiple comparisons. Statistical analyses were performed using IBM SPSS Statistics version 23 (IBM Corporation, Armonk, NY).

## Results

### Patients and procedures

In total, 90 liver tumors in 78 patients were included in this study. Of these 90 tumors, 45 in 35 patients (27 men, median age 67 years, IQR 15 years) were HCCs with a mean diameter of 25.0 mm (SD 9.6 mm), ranging from 6 to 55 mm, and 45 in 43 patients (26 men, median age 64 years, IQR 12 years) were CRLMs with a mean diameter of 22.5 mm (SD 10.6 mm), ranging from 7 to 60 mm. In total, 14 tumors between 30 mm and 40 mm in size, and four larger than 40 mm were included. These larger tumors were treated with thermoablation because in these patients no other form of therapy was available. Seventeen patients had received prior systemic chemotherapy, with a mean of 1.1 years between chemotherapy and ablation. None of the tumors had been treated with transarterial (chemo)embolization.

All 90 tumors were treated in 80 sessions. In 14 tumors (15.5%) the ablation was incomplete. The median ablation zone volume for all tumors was 55.7 mL (IQR 55.9 mL). The interobserver correlation for volumetry of the ablation zone was high (ICC 0.994, 95% confidence interval 0.990–0.996, *p* < 0.001).

#### Comparison of devices

Table 1 shows baseline patient and tumor variables for each ablation device. In general, the median ablation time was longer with RFA than with MWA (*T*_RFA_ = 48 min, *T*_MWA_A_ = 14 min, *T*_MWA_B_ = 20 min; *p* < 0.001). Also, the median applied energy was higher with RFA (*E*_RFA_ = 294.0 kJ, *E*_MWA_A_ = 111.0 kJ, *E*_MWA_B_ = 111.0 kJ; *p* = 0.001). Ablation zone volumes, number of needle positions, tumor volume percentages and numbers of incomplete ablations were approximately similar for all devices. The ablated tumor volume as a percentage of the total ablated volume was remarkably low, with a median value of 11.2% (IQR 5.1–23.1%). *R*(AZ:E) was lower with RFA than with MWA (*R*_RFA_ = 0.17, *R*_MWA_A_ = 0.66, *R*_MWA_B_ = 0.46; *p* < 0.001). The ratios for MWA devices A and B were not significantly different (*p* = 0.344).

**Table 1:** Patient and tumor characteristics

	RFA	MWA_A	MWA_B	*p* value
*R*(AZ:E) (mL/kJ)	0.17 (0.14–0.26)	0.66 (0.37–0.86)	0.46 (0.37–0.77)	<0.001
Number of lesions	30	30	30	
Patients	24	28	26	
Gender (male/female)	19/5	19/9	15/11	0.072
Age (years)	64.3 (7.7)	66.4 (11.2)	65.8 (12.1)	0.741
Lesion diameter (mm)	21.3 (7.2)	25.8 (12.6)	24.1 (9.8)	0.380
Ablation time (min)	48 (21–75)	14 (4–23)	20 (11–29)	<0.001
Applied energy (kJ)	294.0 (83.5–504.5)	111.0 (38.1–183.9)	111.0 (52.9–169.2)	0.001
Ablation volume (mL)	49.4 (23.8–75.2)	63.9 (35.9–92.0)	69.0 (25.6–112.4)	0.364
Needle positions (*n*)	4 (1)	4 (5)	3.5 (2)	0.535
Tumor percent of total ablation zone volume	9.4 (3.4–15.4)	14.0 (3.0–25.0)	11.5 (1.5–21.5)	0.516
Previous systemic therapy	7	5	5	0.611
Incomplete ablations after 1 week follow-up (*n*)	4	7	3	0.118

Normally distributed data are presented as means (SD) and non-normally distributed data as medians (Q1–Q3)

R*(AZ:E)* ratio of ablation zone volume in milliliters to applied energy in kilojoules

With RFA, the needle array size was correlated with *R*(AZ:E) with a Spearman’s correlation coefficient of −0.415 (*p* = 0.023; i.e. larger needle arrays result in a lower *R*(AZ:E). The median needle array sizes were similar for HCC and CRLM: 3.5 cm and 3.5 cm, respectively (*p* = 0.337).

Prior systemic therapy did not have a significant effect on *R*(AZ:E) for any of the devices. With RFA, the median (IQR) *R*(AZ:E) values of CRLMs in patients who did and did not receive prior systemic therapy were 0.14 (0.10–0.20) and 0.18 (0.15–0.23; *p* = 0.083), respectively. With MWA_A, median *R*(AZ:E) values were 0.34 (0.21–0.80) and 0.38 (0.34–0.56; *p* = 0.713), and with MWA_B were 0.35 (0.17–0.75) and 0.43 (0.37–0.62; *p* = 0.391), respectively.

### Comparison of HCC with CRLM

Table 2 shows the clinicopathological and procedural characteristics separately for HCC and CRLM, stratified according to the type of ablation device used. The tumor diameter, number of peritumoral vessels, needle positions and incomplete ablations did not differ between the tumor types, for any of the devices.

**Table 2:** Clinicopathological and procedural characteristics of HCC and CRLM according to ablation device

	RFA			MWA_A			MWA_B		
	HCC	CRLM	*p* value	HCC	CRLM	*p* value	HCC	CRLM	*p* value
*R*(AZ:E) (mL/kJ)	0.22 (0.14–0.45)	0.15 (0.14–0.22)	0.110	0.81 (0.61–1.07)	0.43 (0.35–0.61)	0.001	0.67 (0.41–0.85)	0.43 (0.35–0.61)	0.040
Tumor diameter (mm)	23.2 (5.6)	19.4 (8.2)	0.227	27.9 (12.0)	23.7 (13.2)	0.377	23.8 (10.1)	24.5 (9.9)	0.722
Peritumoral vessels >3mm	3	5	0.682	6	9	0.466	7	3	0.245
Ablation time (min)	40 (22)	76 (38.2)	0.007	13 (9)	21 (14)	0.052	24 (11)	25 (15)	0.631
Applied energy (kJ)	222.5 (215.5)	466.7 (300.4)	0.022	98.2 (62.8)	182.4 (140.7)	0.043	127.1 (73.1)	143.7 (87.1)	0.817
Needle positions (*n*)	3.3 (1.1)	4.2 (2.3)	0.140	4.3 (2.9)	5.2 (3.6)	0.476	3.7 (1.5)	4.0 (2.4)	0.618
Ablation zone volume (mL)	31.6 (13.5–51.7)	54.7 (33.2–67.2)	0.050	69.5 (42.6–96.4)	57.2 (25.8–88.6)	0.548	81.6 (46.6–116.7)	53.3 (21.1–85.6)	0.134
Tumor percent of ablation zone volume	15.8 (8.2–22.8)	5.8 (2.8–8.8)	<0.001	18.0 (6.0–30.0)	10.9 (4.4–17.4)	0.178	5.8 (4.6–7.0)	14.9 (13.9–15.9)	0.412
Incomplete after 1 week of follow-up (*n*)	2	2	1.000	3	4	0.666	1	2	0.543

Normally distributed data are presented as means (SD) and non-normally distributed data as medians (Q1–Q3)

*RFA* radiofrequency ablation, *MWA* microwave ablation, *HCC* hepatocellular carcinoma, *CRLM* colorectal liver metastasis, R*(AZ:E)* ratio of ablation zone volume to applied energy

**Fig. 1:**
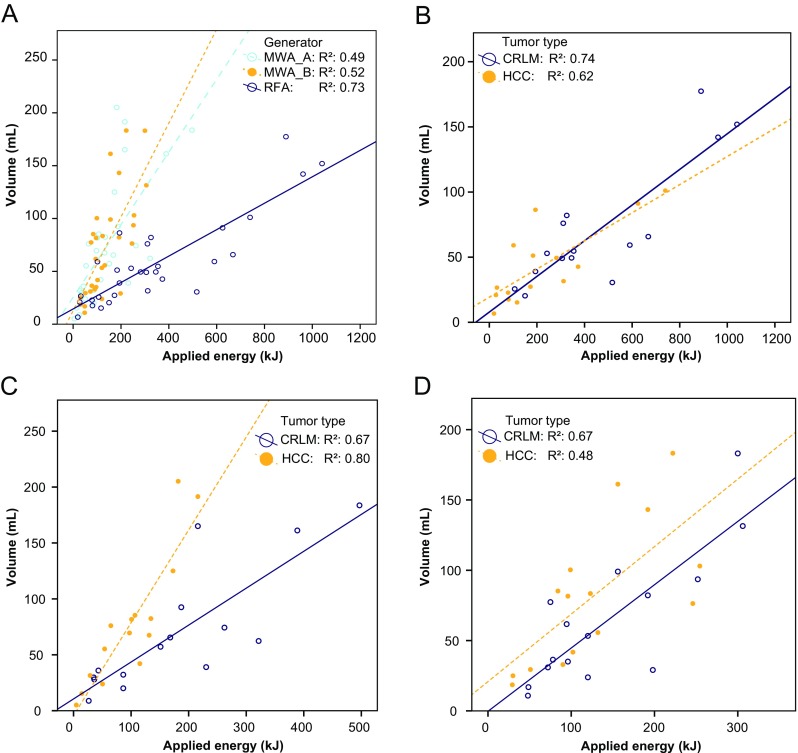
Regression analysis of the relationships between applied energy and (**a**) ablation zone volume for the three devices, (**b**) ablation zone volume obtained with the RFA device (Boston Scientific Corp.) grouped by tumor type, (**c**) ablation zone volume obtained with the MWA device A (Microsulis Medical) grouped by tumor type, and (**d**) ablation zone volume obtained with the MWA device B (Covidien) grouped by tumor type (*CRLM* colorectal liver metastasis, *HCC* hepatocellular carcinoma)

With RFA, *R*(AZ:E) did not differ significantly between tumor types (*R*_HCC_ = 0.22, *R*_CRLM_ = 0.15; *p* = 0.110). With both MWA devices, *R*(AZ:E) was higher for HCC than for CRLM: MWA_A, *R*_HCC_ = 0.81, *R*_CRLM_ = 0.43 (*p* = 0.001), and MWA_B, *R*_HCC_ = 0.67, *R*_CRLM_ = 0.43 (*p* = 0.040). Thus, MWA generates larger ablation zone volumes per kilojoule in HCC than in CRLM. Figure 1 shows the applied energy in relation to the ablation zone volume as scatter plots grouped by generator, and grouped by tumor type according to generator.

**Fig. 2:**
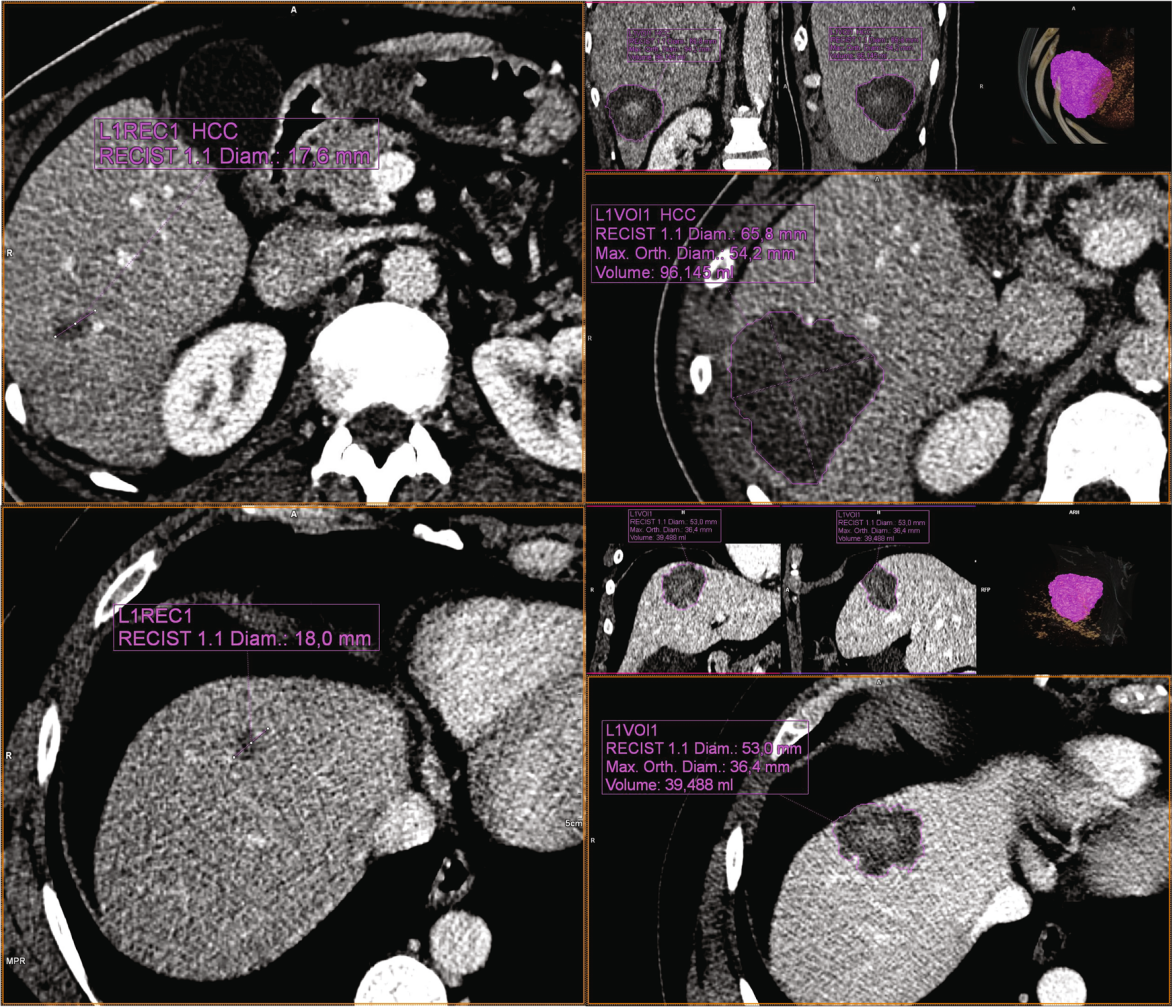
*Left* Preprocedural portal venous phase contrast enhanced CT images of HCC in segment 6 (*top*) and CRLM in segment 4 (*bottom*) in two patients who had not received systemic therapy or transarterial (chemo)embolization. Using MWA device B, 96 kJ (100 W for 8:00 min × 2) and 96 kJ (100 W for 6:00 min, 100 W for 10:00 min) were applied to the HCC and CRLM, respectively, with 16 mm and 14 mm between the two positions of the ablation center of the antenna, so overlap was approximately similar. *Right* Resulting ablation zones after segmentation on the 1-week follow-up portal venous phase contrast-enhanced CT images using the MM Oncology package (*syngo*.via; Siemens, Erlangen, Germany), with ablation zone volumes of 96 mL and 39 mL, resulting in energy deposition ratios of 1.00 mL/kJ and 0.41 mL/kJ for HCC and CRLM, respectively. After 6 months of follow-up, the HCC showed no sign of recurrence, whereas a PET scan of the CRLM showed activity at the dorsal side of the ablation zone, for which re-ablation was performed

Example images of an HCC and a CRLM prior to ablation with MWA_B and of the resulting ablation zone volumes are shown in Fig. 2. In both these cases, 96 kJ of energy was applied, yet the resulting ablation zone volume in the HCC was more than twice that in the CRLM.

There were no significant correlations between heat-sink and tumor type for RFA, MWA_A and MWA_B (*p* = 0.682, *p* = 0.466, *p* = 0.245, respectively). There were no significant differences in *R*(AZ:E) between ‘high’ and ‘low’ heat-sink tumors with RFA (*R*_high_ = 0.20, IQR 0.15–0.24, *R*_Low_ = 0.16, IQR 0.13–0.28, *p* = 0.743). With MWA_A, *R*(AZ:E) was significantly lower for ‘high’ heat-sink tumors (*R*_high_ = 0.39, IQR 0.33–0.76, *R*_low_ = 0.83, IQR 0.47–1.06, *p* = 0.016), but with MWA_B there was no significant difference in *R*(AZ:E) between ‘high’ and ‘low’ heat-sink tumors (*R*_high_ = 0.55, IQR 0.40–0.89, *R*_low_ = 0.46, IQR 0.37–0.65, *p* = 0.422). Thus, the ablation zones created by MWA_A were smaller in the presence of peritumoral vessels ≥3 mm than those created by the other two devices which seemed to be less influenced by the presence of such vessels.

## Discussion

The purpose of this study was to evaluate the relationship between the amount of applied energy and the resulting ablation zone volume in liver tumors for RFA and MWA devices. To this end *R*(AZ:E) was calculated for each procedure. With RFA, *R*(AZ:E) was similar in HCC and in CRLM. With both MWA devices, *R*(AZ:E) was higher in HCC than in CRLM (about twofold higher with MWA_A and about 50% higher with MWA_B).

The HCCs selected for this study were all in patients with a cirrhotic liver, whereas the patients with CRLMs had an otherwise healthy liver. The difference in the treatment effects observed between the two tumor types may have been the result of differences in tumor characteristics and in the surrounding liver parenchyma associated with the underlying liver disease. Probably, all of these factors contributed to the differences measured. The differences measured may also be associated with underlying differences in electrical properties (relative permittivity and electrical conductivity) and/or thermal properties (thermal conductivity, specific heat capacity, density and nominal blood perfusion rate).

Deshazer et al. recently investigated the effect of these properties on the resulting MWA zone using a two-compartment (tumor and surrounding liver parenchyma) computer model [11]. They concluded that the ablation zone volume was minimally affected by the tissue characteristics ; the predominant factors influencing the ablation zone volume were differences in thermal conductivity and hepatic perfusion of the surrounding liver parenchyma. In our study, most of the ablated tissue volume was liver parenchyma and less than 20% consisted of tumor. This seemingly low ratio can be explained by the example that the volume of a spherical 10-mm tumor would only be 12.5% of the total ablation zone volume if a perfect 5-mm safety margin was created around the tumor. This low tumor percentage supports the findings of Deshazer et al. indicating that the main factor influencing the difference in treatment effects between the tumor types is a difference in tissue characteristics between normal and cirrhotic liver parenchyma.

Another variable could be the difference in total liver blood flow between normal livers (with CRLM) and cirrhotic livers (with HCC). Although in some studies an increase in total liver blood flow has been found in cirrhotic livers [12], in most studies cirrhotic liver parenchyma has been found to be less perfused than normal liver parenchyma [13, 14]. According to the model, a decrease in liver perfusion from 18 kg m^−^1s^−^1 (normal) to 11 kg m^−^1s^−^1 (cirrhotic) would result in an increase in ablation zone volume of 37% [11]. This corresponds approximately to our results with the MWA devices.

Differences in energy deposition between HCC and CRLM could also be caused by the pseudocapsule of HCCs. This pseudocapsule has been considered as a reason for the occurrence of a hypothetical “oven effect”, that is higher temperatures inside the tumor, but lower temperatures outside because of the low thermal conductivity of the capsule. With RFA, it remains to be seen how much heat this capsule actually helps to contain. With MWA, this oven effect is less likely to occur, as MWA does not rely on thermal conductivity in contrast to RFA. More detail about the technical differences between MWA and RFA in HCC can be found in a review by Poggi et al. [15]. Since the observed difference in ablative effects between HCC and CRLM in this study occurred only with MWA, it is not likely that the oven effect was a significant contributor.

With RFA, the size of the umbrella array significantly affected the ratio of energy deposition. Larger arrays resulted in lower *R*(AZ:E). This might have been because smaller needle arrays have a better energy deposition, or because larger arrays have relatively more overlap when performing ablation at multiple locations, and thereby lose energy deposition efficiency. For both tumor types the median RFA antenna size was similar, so it is unlikely that this contributes to how tumor type affects the ratio of energy deposition.

To our knowledge, there is only one clinical study comparing the correlation between applied energy and ablation zone size with MWA in HCCs and CRLMs [16]. That study generated conflicting results: for shorter ablation times they found no difference in effect, and for longer ablation times they found smaller ablation diameters in HCC than in metastases. They did not, however, measure the volume of the ablation zone, but only diameters. These were all procedures with a single insertion and energy delivery. No overlapping ablations were included, whereas most of the procedures in this study were performed with overlapping ablations, which might be a reason for the observed differences in outcome.

Lu et al. investigated the effect of the presence of a flow heat-sink and found that the presence of large (>3 mm) peritumoral vessels resulted in a higher ablation site malignancy recurrence rate after RFA in liver tumors [8]. The presence of such a heat-sink can be expected to affect the amount of energy required to achieve a certain ablation volume. In our study, this effect was demonstrated with MWA device A which produced a lower *R*(AZ:E) in the tumors with peritumoral vascularity than in the tumors without peritumoral vascularity. In the RFA and MWA_B groups there was no difference in *R*(AZ:E) between these types of tumor. MWA_B utilizes thermal, field, and wavelength control to produce spherical ablation zones, which might have contributed to the apparent insensitivity to the presence of a heat-sink [17]. The fact that no heat-sink effects were observed in the RFA group was somewhat unexpected and may have been because only 8 of 30 tumors in that group were classified as potentially ‘high’ heat-sink tumors.

Although both MWA devices operated in the 2.45 GHz frequency range, their level of power output differed: MWA_A generated more power than MWA_B. It is unclear how much of this power was actually transferred into the tissue because this is also affected by the energy reflected by the cable and antenna. However, with MWA_A, the transferred power was probably higher and the duration was certainly shorter than with MWA_B. Studies investigating the effect of differences in level of power output have generally compared 915 MHz and 2.45 GHz devices [18, 19]. It is therefore unclear how this might have affected the observed differences between the two MWA devices.

There were some limitations to this study. Often a roundness index is used to quantify the quality of the ablation properties of a system [20]. This is because spherical ablation zones are easier to predict and can potentially cover a tumor better than ovoid ablation zones. In this study, however, 95% of the tumors were treated at multiple needle positions which meant that a roundness index of the entire ablation zone volume would not have been an accurate measure. This also means that the amount of overlap could have contributed to differences in the volume–energy relationship, because the electrical and thermal properties of tissues change after ablation. We were not able to determine the amount of overlap in the procedures nor to compensate for this. Despite this, mean lesion diameter, the number of needle positions, and the ablated tumor volume expressed as a percentage of the total volume were not different for the three devices or between tumor types for the MWA devices. Therefore, it is not likely that the amount of overlap was any different between these groups either. Additionally, the nongeometric configuration of the ablation zones was taken into account by a precise volume evaluation of the ablation zone.

Finally, histology of adjacent liver parenchyma was not performed, since it is not clinical practice to perform biopsies, either of the tumor or of the liver, because diagnosis of both is mainly determined based on the medical history of the patient, laboratory results and radiological imaging.

In conclusion, with the MWA devices we found a difference in the ablation zone volume per kilojoule of energy applied between tumor types. Because HCCs require less energy to achieve a certain ablation volume, CRLMs must be treated by creating multiple ablations with plenty of overlap, or at a relatively high power level or for a longer time. These data clearly demonstrate that the manufacturers’ algorithms based solely on power level and duration of application need to be adapted to the type of tumor in its specific environment. Therefore, it is critical to verify the dimensions of the ablation zone early after the procedure using contrast-enhanced CT or preferably contrast-enhanced magnetic resonance imaging.

## Abbreviations

CRLM: Colorectal Liver Metastasis
HCC: Hepatocellular Carcinoma
ICC: Intraclass Correlation
IQR: Interquartile Range
MWA: Microwave Ablation
*R*(AZ:E): Ratio of Ablation Zone Volume in Milliliters to Applied Energy in Kilojoules
RFA: Radiofrequency Ablation
SD: Standard Deviation
